# Sex-Stratified Single-Cell RNA-Seq Analysis Identifies Sex-Specific and Cell Type-Specific Transcriptional Responses in Alzheimer’s Disease Across Two Brain Regions

**DOI:** 10.1007/s12035-021-02591-8

**Published:** 2021-10-20

**Authors:** Stella A. Belonwu, Yaqiao Li, Daniel Bunis, Arjun Arkal Rao, Caroline Warly Solsberg, Alice Tang, Gabriela K. Fragiadakis, Dena B. Dubal, Tomiko Oskotsky, Marina Sirota

**Affiliations:** 1 Bakar Computational Health Sciences Institute, University of California San Francisco, 490 Illinois St, San Francisco, CA 94143 USA; 2 Pharmaceutical Sciences and Pharmacogenomics Graduate Program, University of California San Francisco, San Francisco, CA USA; 3 CoLabs, University of California, San Francisco, San Francisco, CA USA; 4 Bakar ImmunoX Initiative, University of California, San Francisco, San Francisco, CA USA; 5 Department of Pathology, University of California San Francisco, San Francisco, CA USA; 6 Bioengineering Graduate Program, University of California San Francisco, San Francisco, CA USA; 7 Department of Medicine, Division of Rheumatology, University of California, San Francisco, San Francisco, CA USA; 8 Biomedical Sciences Graduate Program, University of California, San Francisco, San Francisco, CA USA; 9 Neurosciences Graduate Program, University of California, San Francisco, San Francisco, CA USA; 10 Department of Neurology and Weill Institute for Neurosciences, University of California, San Francisco, San Francisco, CA 94158 USA; 11 Department of Pediatrics, University of California San Francisco, San Francisco, CA USA

**Keywords:** Alzheimer’s disease, Sex differences, Single-cell, RNA sequencing

## Abstract

Alzheimer’s disease (AD) is a pervasive neurodegenerative disorder that disproportionately affects women. Since neural anatomy and disease pathophysiology differ by sex, investigating sex-specific mechanisms in AD pathophysiology can inform new therapeutic approaches for both sexes. Previous bulk human brain RNA sequencing studies have revealed sex differences in dysregulated molecular pathways related to energy production, neuronal function, and immune response; however, the sex differences in disease mechanisms are yet to be examined comprehensively on a single-cell level. We leveraged nearly 74,000 cells from human prefrontal and entorhinal cortex samples from the first two publicly available single-cell RNA sequencing AD datasets to perform a case versus control sex-stratified differential gene expression analysis and pathway network enrichment in a cell type-specific manner for each brain region. Our examination at the single-cell level revealed sex differences in AD prominently in glial cells of the prefrontal cortex. In the entorhinal cortex, we observed the same genes and networks to be perturbed in opposing directions between sexes in AD relative to healthy state. Our findings contribute to growing evidence of sex differences in AD-related transcriptomic changes, which can fuel the development of therapies that may prove more effective at reversing AD pathophysiology.

**Supplementary Information:**

The online version contains supplementary material available at 10.1007/s12035-021-02591-8.

## Background

Alzheimer’s disease (AD) is an irreversible neurodegenerative disorder that causes progressive memory decline, cognitive deficits, and behavioral changes [1–3]. It is the most common form of dementia and is reaching epidemic proportion as a result of extended life expectancies and increased elderly populations worldwide [4, 5]. It is of high priority to find disease-modifying treatments for AD, as more than five million people are diagnosed with AD currently in the USA, a number estimated to triple by 2050 [6, 7].

Although first described more than a century ago [8], the underlying molecular mechanisms of AD remain elusive [9]. Extensive research efforts reveal that AD is histologically characterized by pathological brain aggregates including extracellular amyloid-β (Aβ) plaques and intracellular tau protein neurofibrillary tangles [10, 11]. Increasing evidence suggests that neuroinflammation and brain dysfunction led by neuronal supporting cells, which include microglia, astrocytes, and oligodendrocytes, could contribute to AD pathophysiology [12, 13]. These pathological features are accompanied by impaired neurotransmitter signaling, dysregulated neuronal metabolism, neuronal loss, and cerebral atrophy [14–16]. Overall, the exact pathogenesis of AD remains uncertain, which hinders the development of effective therapies.

Sex differences have been clinically documented in AD [17, 18], yet the underlying causes for these differences are not well understood. Approximately two thirds of AD diagnoses are in women [19]. In addition to greater longevity in females [20], other biological differences may be responsible for the higher prevalence and accelerated cognitive decline observed in women during disease progression [18, 21, 22]. For instance, a longitudinal study examining a postmortem cohort of about 1500 individuals observed that in the presence of similarly high Aβ burden, females exhibited faster cognitive decline than males [22], suggesting females might be more susceptible to Aβ toxicity. Furthermore, after adjusting for age and education, women had a higher tau tangle density [22, 23]. Among genetic risk factors implicated in AD, the apolipoprotein E (*APOE*) ε4 risk allele has been observed to have a differential influence and increased risk for AD in women compared to men [24, 25]. Sex hormones, especially the decline in hormone levels postmenopause, could also contribute to sex differences in AD progression. For example, after menopause, women experience an abrupt loss of progesterone [26], which was previously shown to be neuroprotective by promoting myelin repair and reducing inflammation [27, 28]. In fact, compared to men, women experience more inflammation-driven symptoms and have an increased risk for autoimmune diseases [29–31]. These findings suggest that investigating sex differences in AD will not only provide insight into deciphering the fundamental biological and mechanistic causes of AD pathogenesis, but also highlight the necessity of developing personalized therapeutic strategies.

Previous studies suggest that cellular and molecular heterogeneity in AD pathogenesis [32, 33] and brain immune cell dysfunction contribute to sex-specific AD pathophysiology [34]; however, sex-specific disease complexity at single-cell resolution is masked in bulk brain RNA sequencing (RNA-Seq) analysis. Recent advances in single-cell RNA-Seq technology and the increasing availability of human transcriptomic datasets present a novel opportunity to examine cell type-specific transcriptional alterations in AD brain pathology. Previously, genomic analyses have been performed on heterogeneous populations of cells, and thus, observed signals represented a combination of the unique characteristics of each individual cell. In the last few years, high-throughput single-cell and single-nucleus techniques have revolutionized the field, allowing for high-dimensional analysis of isolated subpopulations of individual cells and enabling an unprecedented level of granularity in characterizing gene expression changes in disease models. Researchers now have the opportunity to address key challenges barring advancements in the field of AD research by studying sample heterogeneity and transcriptomic signatures that are specific to the disease-relevant cells. This approach allows mapping the spectrum of neuronal and other relevant cell types, describing their cell-specific signaling pathways in disease and ascertaining which of these features might explain the sex- or genotype-specific disease etiology in AD.

In recent years, two single-nucleus RNA-Seq (snRNA-Seq) datasets were generated from the prefrontal [35] and entorhinal [36] cortices of age- and sex-matched human AD patients and cognitively normal controls. For the prefrontal cortex dataset, Mathys and colleagues performed differential expression analysis on single-cell transcriptomic results across 48 individuals of varying degrees of AD pathology and reported on the general sexual dimorphic transcriptional response to AD pathology; however, they did not extensively examine sex-specific differentially expressed genes (DEGs) in the individual brain cell types or delineate any subsequent sex-specific molecular pathway enrichments in AD. Similar to the Mathys analysis, Grubman and colleagues analyzed single-nuclei transcriptomes sequenced from the entorhinal cortex of 12 age- and sex-matched human AD patients and controls. Besides investigating the likelihood of sex as a covariate factor for DEG variance observed, no sex difference analysis was performed in this study.

Understanding gene expression changes unique to each sex provides opportunities to decipher molecular underpinnings that differentially contribute to AD in males and females. In this study, we leveraged these two snRNA-Seq datasets to characterize sex-stratified cell type-specific gene expression perturbations in AD and to identify sex-specific disease-associated cellular pathways as potential precision therapeutic targets. In both brain regions, we identified sex-specific disease changes primarily in glial cells and observed samples to cluster by sex when examining gene expression changes in AD compared to controls. Our findings will be of fervent interest to the field in studying differing vulnerabilities between sexes in AD.

## Methods

### Study Cohorts

**Fig. 1:**
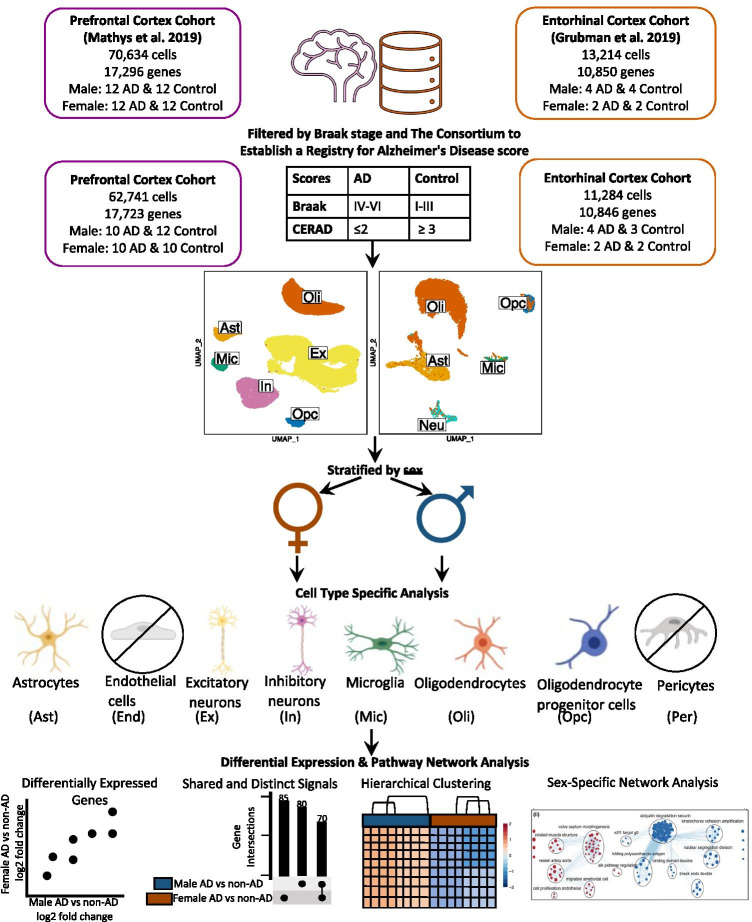
Workflow for cohort sample definition and sex-stratified cell type-specific differential gene expression and functional enrichment. AD and non-AD cells were determined based on tau (Braak) and amyloid-β plaque (CERAD) burdens. Cell types were identified, and AD versus non-AD differential expression and pathway network enrichment analyses were performed separately for each sex in each cell type

The prefrontal cortex cohort comprised age- and sex-matched samples from 24 males and 24 females with varying degrees of AD pathology. We reclassified samples based on tau and amyloid-β (Aβ) plaque burden, using Braak clinical staging and Consortium to Establish a Registry for Alzheimer’s Disease (CERAD) scores [37], respectively. We defined cases as individuals with severe tau deposition (Braak ≥ IV) and high Aβ load (CERAD ≤ 2), and non-AD controls as individuals with low tau (Braak ≤ III) and low Aβ load (CERAD ≥ 3). For our sex-stratified analysis, we focused on 20 cases (10 females, 10 males) and 22 controls (10 females, 12 males) (Fig. 1, Table 1, Supplementary Table 1).

**Table 1:** Prefrontal cortex cohort

Characteristic	AD	Control
*n* total	20	22
Age, mean (SD)	85.3 (4.7)	84.8 (4.5)
Sex, *n* (%)		
Female	10 (50.0)	10 (45.5)
Male	10 (50.0)	12 (54.5)
APOE, *n* (%)		
2/3	2 (10.0)	7 (31.8)
3/3	9 (45.0)	14 (63.6)
3/4	6 (30.0)	1 (4.5)
4/4	3 (15.0)	0 (0.0)

The entorhinal cortex cohort consisted of age-matched 6 (2 females, 4 males) AD patients and 6 (2 females, 4 males) control subjects, as indicated by Grubman et al. All cases had a history of AD, while controls had no history of AD or cognitive impairment, as reported by treating general practitioners. For pathological scores used in categorizing samples, Braak staging scores were provided only for cases, and amyloid pathology information was provided for all samples using the following categories: “Numerous diffuse and neuritic Aβ plaque,” “Occasional diffuse plaque in cortex,” and “None.” To use the same scoring system for identifying cases and controls in both datasets, we used criteria from the Rush Alzheimer’s Disease Center clinical codebook provided with the prefrontal cortex dataset to convert these measures of neuritic plaques into CERAD scores of 1 (Definite), 3 (Possible), and 4 (No AD), respectively. We excluded one control male sample with the APOE2/4 genotype. For our sex-stratified analysis, we focused on 6 cases (2 females, 4 males) and 5 controls (2 females, 3 males) (Fig. 1, Table 2, Supplementary Table 2).

**Table 2:** Entorhinal cortex cohort

Characteristic	AD	Control
*n* total	6	5
Age, mean (SD)	78.9 (8.5)	75.1 (5.7)
Sex, *n* (%)		
Female	2 (33.3)	2 (40.0)
Male	4 (66.7)	3 (60.0)
APOE, *n* (%)		
2/3	0 (0.0)	0 (0.0)
3/3	1 (16.7)	4 (80.0)
3/4	3 (50.0)	1 (20.0)
4/4	2 (33.3)	0 (0.0)

### Single-Cell Data Processing, Cell Type Identification, and Batch Correction

Data processing and analysis were performed separately for each dataset with R [38] version 4.0.0 (April 24, 2020) via RStudio [39], using Seurat [40] (v3.1.5). Visualizations were created with BioRender (https://biorender.com/) (Fig. 1), dittoSeq (v1.0.2) (https://github.com/dtm2451/dittoSeq/), a package for analysis and visualization of bulk and single-cell transcriptomic data in a color blind friendly manner, ggplot2 [41], and UpsetR [42]**.**

#### Prefrontal Cortex

Seurat’s Read10X function was used to generate a count data matrix using the filtered count matrix of 17,296 genes and 70,634 cells, gene names, and barcode files provided by 10X. A Seurat object was created with the count data matrix and metadata and filtered to keep genes present in at least 3 cells and cells meeting cohort selection criteria of at least 200 genes. Log normalization was performed using Seurat’s *NormalizeData* function with a scale factor of 10,000, and highly variable features were identified using Seurat’s *FindVariableFeatures*, returning 3188 features, as specified in the original paper. The data matrix was then scaled using Seurat’s *ScaleData* function with *nCount_RNA* regressed out, and dimensionality reduction through Uniform Manifold Approximation and Projection (UMAP) was performed with the appropriate dimensions selected based on the corresponding principal component analysis (PCA) elbow plot. UMAP plots confirmed that there were no confounding variables (Supplementary Fig. 1).

To identify cell types, following similar steps as Grubman and colleagues [36], we applied Seurat’s *AddModuleScore* function to list of 200 brain cell type markers from the BRETIGEA [43] package to identify each cell type. Cell types assessed included astrocytes, neurons, microglia, oligodendrocytes, oligodendrocyte progenitor cells (OPCs), pericytes, and endothelial cells. Cells with the highest score across brain cell type markers were labeled the corresponding cell type, and if the highest and second highest scores were within 20%, cells were deemed hybrids and excluded from further analysis. We further confirmed successful cell type identification by assessing homogeneity and separation of clusters in UMAP plots and by examining expression of top marker genes across cell types. While cell type identification with BRETIGEA package’s cell type markers was comparable to the original paper’s identification, we found the original paper’s cell types more comprehensive as it distinguished excitatory from inhibitory neurons. Thus, we used the original paper’s cell type labels for the further analysis (Supplementary Table 3). Due to low cell counts, we did not analyze pericytes and endothelial cells. The final Seurat object contained 17,723 genes and 62,741 cells.

#### Entorhinal Cortex

We acquired a filtered raw expression matrix of 10,850 genes and 13,214 cells, which was originally composed of 33,694 genes and 14,876 cells and filtered as described by Grubman and colleagues. Briefly, genes without any counts in any cells were filtered out. A gene was kept in the analysis if two or more transcripts were present in at least ten cells. The 100 postmortem interval (PMI)-associated genes were removed from further analysis. Cells outside the 5th and 95th percentiles with respect to the number of genes detected and the number of UMIs and cells with more than 10% of their UMIs assigned to mitochondrial genes were filtered out. The matrix was normalized by the Seurat pipeline (scale factor of 10,000) and ScaleData was used to center the gene expression resulting in a filtered matrix consisting of 10,850 genes and 13,214 cells. A Seurat object was created and consisted of genes in at least 3 cells and cells with at least 200 genes. Normalization was performed using Seurat’s *SCTransform* [44] method, and Seurat’s integration workflow was performed to correct the confounded batches introduced by the original study’s experimental design.

Dimensionality reduction was performed using values from the integrated assay to assess successful batch correction (Supplementary Fig. 1). Using the method for cell type identification described for the former cohort, we identified astrocytes, endothelial cells, neurons, microglia, oligodendrocytes, and OPCs. We further confirmed successful cell type identification by assessing homogeneity and separation of clusters in UMAP plots. Due to limitations in the number of cells, we excluded endothelial cells from further analyses. The final Seurat object contained 10,846 genes and 11,284 cells (Supplementary Table 4).

### Cell Type-Specific Sex-Stratified Differential Expression Analysis

To generate molecular signatures relative to sex in each cell type, we used the Limma [45, 46] package’s voom [47] pipeline for RNA-Seq. For the prefrontal and entorhinal cortices, we performed a sex-stratified analysis including *APOE* genotype as a covariate. For the entorhinal cortex cohort, while we integrated batches in our preprocessing, we were not able to include batch as a covariate, as its collinearity did not allow for an appropriate model fit.

After the design formulas were established, the DGEList object was created from a matrix of counts extracted from the corresponding Seurat objects. To improve the accuracy of mean–variance trend modeling and lower the severity of multiple testing correction, lowly expressed genes were filtered out using edgeR’s FilterByExpr function with default parameters. Normalization was performed with trimmed mean of *M* values with singleton pairing (TMMwsp), followed by voom, model fitting with a contrast matrix of each defined case–control comparison, and empirical Bayes fitting of standard errors. We determined differentially expressed genes (DEGs) as those with a Benjamini-Hochberg (BH)-corrected *p* value less than 0.05 and an absolute log2 fold change (LFC) greater than 0.25. We then examined AD compared to control gene expression changes in all cell types of each sex using pairwise gene expression plots, violin plots of gene expression, hierarchical clustering of samples using AD compared to control pseudobulk cell type gene expression, and Upset plots, which prioritized labeling DEGs with more overlaps across the groups compared.

### Pathway Analysis

We performed an overrepresentation analysis of DEGs from the cell type-specific sex-stratified analysis of cells from the prefrontal and entorhinal cortex using g:Profiler [48], a web tool that performs functional enrichment analysis from a given gene list. We queried DEGs split by upregulated and downregulated expression and selected enriched pathways with a BH-adjusted *p* value cutoff of 0.05. In addition to Gene Ontology cellular components, biological processes, and molecular functions, our enrichment analysis also provided pathways from the Human Protein Atlas, Human Phenotype Ontology, KEGG, Reactome, and Wiki pathways.

### Network Visualization of Enrichment Results

We followed a previously established protocol [49] for network enrichment analysis on pathway results derived from our cell type-specific DEGs. Briefly, pathway results were imported into the Cytoscape visualization application, EnrichmentMap. Then, redundant and related pathways were collapsed into single biological themes using the AutoAnnotate Cytoscape application.

## Results

### Sample Classification and Analytic Workflow

Samples were categorized into cases and controls based on tau tangle and Aβ plaque burdens, using Braak clinical staging and CERAD scores [37], respectively (AD: Braak stage EM C; CERAD score ge EMcontrol: Braak stage ≤ III; CERAD score e EM CSL_ resulted in snRNA-Seq datasets containing 17,723 genes expressed by 62,741 cells from the prefrontal cortex cohort (Table 1, Supplementary Table 1) and 10,846 genes expressed by 11,284 cells from the entorhinal cortex cohort (Table 2, Supplementary Table 2), which were acquired from different sets of individuals (Fig. 1). In both brain regions, a sex-stratified differential gene expression (DGE) analysis was performed comparing AD cases to controls, with *APOE* genotype as a covariate, in astrocytes (Ast), microglia (Mic), excitatory neurons (Ex), inhibitory neurons (In), undifferentiated neurons (Neu), oligodendrocytes (Oli), and OPCs (Supplementary Tables 3 and 4). For the entorhinal cortex cohort, data integration was performed and *APOE* genotype was included as a sole covariate in our DGE analysis to account for batch effects and avoid collinearity in our model. DEGs were determined using a BH-adjusted *p* value < 0.05 and absolute LFC > 0.25 as cutoffs. DEGs were passed as inputs for pathway enrichment analysis, which provided pathways to be used as inputs for subsequent network analysis. We examined gene expression and pathway networks in AD versus neurotypical cells to identify cell type- and brain region-specific and non-specific differences based on sex.

### Sex-Stratified DGE Analysis in the Prefrontal Cortex Reveals Sex-Specific Disease-Related Changes in Glial Cell Types

**Fig. 2:**
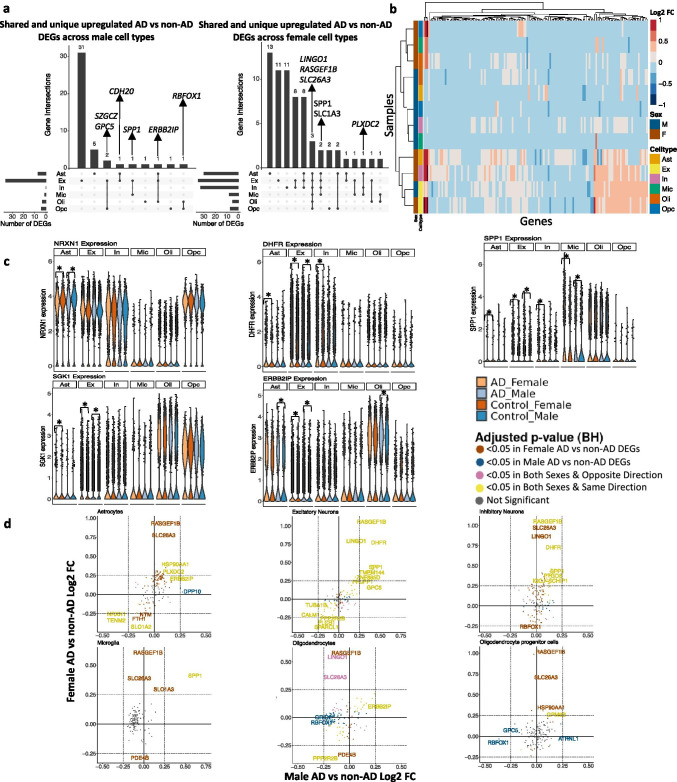
Sex-stratified cell type-specific differential gene expression signatures in the prefrontal cortex. **a**. Upset plots indicating intersections of AD versus non-AD DEGs (BH-adjusted *p* value < 0.05 and absolute LFC > 0.25) across cell types. Rows correspond to cell types. The bar chart shows the number of single and common sets of DEGs across cell types. Single filled dots represent a unique set of DEGs for the corresponding cell type. Multiple filled black dots connected by vertical lines represent common sets of DEGs across cell types. **b**. LFC scores of all genes in the DE analysis clustered by cell type and sex. **c**. *LINGO1*, *PLXDC2*, *SPP1*, *RBFOX1*, and *ERBB21P* expressions. Asterisks represent meeting both significance and absolute LFC thresholds. Colors correspond to sex and AD status. **d**. Pairwise DEG plots of DEGs in male and female samples using LFC scores. Genes shown are significant and have a LFC > 0.25 in at least one sex. Colors indicate significance level of DEGs and whether DEGs are unique or shared by both sexes

Leveraging data from Mathys et al., from our sex-stratified DGE analysis, we identified DEGs meeting significance and LFC thresholds (Table 3) in all cell types except male inhibitory neurons when comparing AD to non-AD (Supplementary Table 5). We identified 73 DEGs across all cell types in the prefrontal cortex (Table 3, Supplementary Table 5). Of these DEGs, 36 were shared in both sexes, while 8 and 29 were specific to AD compared to control males and females, respectively. We also observed more shared DEGs in AD case versus control female signatures versus male signatures across the cell types (Fig. 2a), which is consistent with previous bulk tissue analysis [34]. Some of the DEGs that overlap most across cell types within one sex or across sexes include *LINGO1*, a negative regulator of myelination [50, 51], which we found upregulated in all AD compared to control female cell types; *SLC1A3*, which encodes excitatory amino acid transporter 1 that transports glutamate in the synaptic cleft [52] and was perturbed in all female AD compared to control cell types except oligodendrocytes and OPCs; and *SPP1*, a protein involved in neuroinflammation also known as osteopontin [53] that we observed to be upregulated in AD versus control samples of both female and male excitatory neurons and microglia, as well as female astrocytes and inhibitory neurons. Also, clustering samples by AD compared to control pseudobulk cell type gene expression (Fig. 2b) showed samples to cluster by sex before cell type identity for all cell types except excitatory neurons.

**Table 3:** Number of differentially expressed genes in both sexes per cell type in the prefrontal cortex

	Male		Female	
	Up	Down	Up	Down
Ast	5	2	23	7
Ex	33	3	30	5
In	0	0	32	1
Mic	1	0	6	2
Oli	1	2	3	2
Opc	1	3	7	0

In addition to identifying shared DEGs across cell types and sexes, we also observed a larger range of LFC in the analysis of female AD versus control ([− 0.423, 1.058], median = 0.314) compared to the analysis of male AD versus control ([− 0.370, 0.620], median = 0.343). Within each cell type, we observed DEGs, a number of which are relevant to and have been studied in AD (e.g., *NRXN1* [54], *SPP1* [53], *DHFR* [55], *SGK1* [56], *ERBB2IP* [57]), meeting significance and LFC thresholds. These DEGs are shared by both sexes in AD versus control astrocytes, microglia, and excitatory neurons, with consistent directionality in both sexes (Fig. 2c, d, yellow color; Supplementary Fig. 3). Overall, in the prefrontal cortex, we identified sex-distinct disease-related transcriptomic changes in gene expression primarily among glial cells (Fig. 2d, brown color for female-distinct and blue color for male-distinct).

### Sex-Stratified DGE Analysis in the Entorhinal Cortex Reveals Sex-Specific Disease-Related Changes, Including Opposite Transcriptomic Changes Between Sexes

**Fig. 3:**
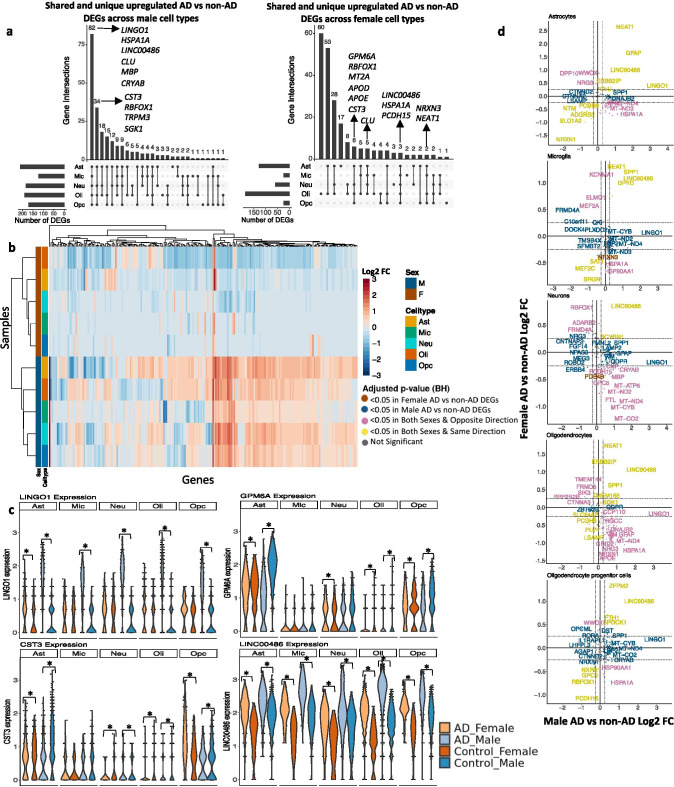
Sex-stratified cell type-specific differential gene expression signatures in the entorhinal cortex. **a**. Upset plots indicating intersections of AD versus non-AD DEGs (BH-adjusted *p* value < 0.05 and absolute LFC > 0.25) across cell types. Rows correspond to cell types. The bar chart shows the number of single and common sets of DEGs across cell types. Single filled dots represent a unique set of DEGs for the corresponding cell type. Multiple filled black dots connected by vertical lines represent common sets of DEGs across cell types. **b.** LFC scores of all genes in the DE analysis clustered by cell type and sex. **c**. *LINGO1*, *GPM6A*, *CST3*, and *LINC00486* expressions. Asterisks represent meeting both significance and absolute LFC thresholds. Colors correspond to sex and AD status. **d**. Pairwise DEG plots of DEGs in male and female samples using LFC scores. Genes shown are significant and have a LFC > 0.25 in at least one sex. Colors indicate significance level of DEGs and whether DEGs are unique or shared by both sexes

Leveraging data from Grubman et al., we identified DEGs (Table 4) comparing AD to non-AD in all cell types stratified by sex. We identified 232 DEGs across all cell types in the entorhinal cortex (Table 4, Supplementary Table 6). Of these DEGs, 211 were shared in both sexes, while 20 and 1 were specific to AD compared to control males and females, respectively. We observed shared DEGs across cell types when comparing AD versus control samples in both sexes (Fig. 3a). Some of the DEGs that overlap most across cell types within one sex or across sexes include *CLU* [9, 58], *HSPA1A* [59], *RBFOX1* [60], and *CST3* [61], which are relevant in AD progression. Clustering of samples by AD compared to control pseudobulk cell type-specific gene expression (Fig. 3b) showed samples to cluster by sex before cell type identity for every cell type and highlighted opposing gene expression patterns based on sex. Indeed, interestingly, 186 of the 211 DEGs shared between male and female AD were regulated in opposite directions with respect to controls, at least in some cell types.

**Table 4:** Number of differentially expressed genes in both sexes per cell type in the entorhinal cortex

	Male		Female	
	Up	Down	Up	Down
Ast	175		19	109
Mic	101	23	9	15
Neu	155	30	13	46
Oli	150	40	22	163
Opc	141	31	13	12

When comparing the magnitude of gene expression changes across sexes in AD versus control samples, we found males to have a greater range of LFCs ([− 2.174, 3.461], median = 0.567) compared to females ([− 1.657, 2.649], median =  − 0.436). We visualized these differences in DEGs such as *LINGO1*, which had a higher fold change difference in male astrocytes (3.415) compared to female astrocytes (0.4); *GPM6A*, which was upregulated in male oligodendrocytes and downregulated in female oligodendrocytes; *CST3*, which was upregulated in male neurons, male oligodendrocytes, and male and female OPCs, and downregulated in female neurons, female oligodendrocytes, and male and female astrocytes; and *LINC00486*, which was upregulated in all cell types of both sexes with an average LFC in males of 1.9 compared to 1.0 in females (Fig. 3c). Generally, directly comparing AD versus control DEGs within each cell type, we not only observe a subset of genes with directionally consistent changes among males and females (Fig. 3d, yellow color; Supplementary Fig. 3), but we also observed numerous changes in opposing directions across sexes (Fig. 3d, pink color; Supplementary Fig. 3) and a higher magnitude of disease-related changes in males compared to females.

### Comparative Analysis Across Brain Regions Reveals More Shared Transcriptomic Sex Differences in the Entorhinal Cortex

**Fig. 4:**
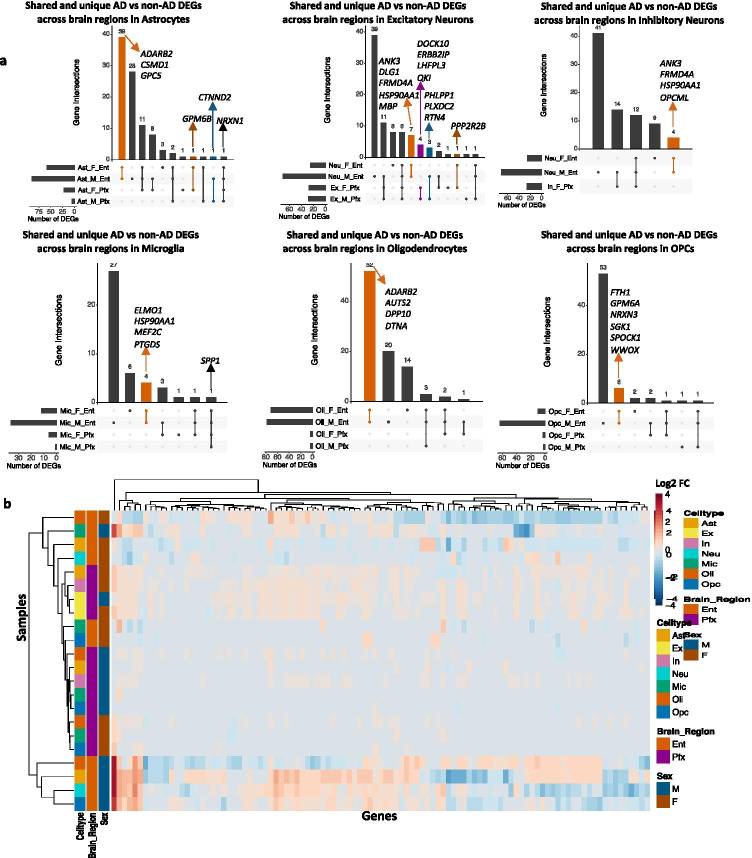
Sex-stratified cell type-specific disease signatures across brain regions. **a**. Upset plots indicating intersections of AD versus non-AD DEGs (BH-adjusted *p* value < 0.05 and absolute LFC > 0.25) within cell types across brain region and sex. Rows correspond to brain region and sex pairings. The bar chart shows the number of single and common sets of DEGs across brain regions and sex. Single filled dots represent a unique set of DEGs for the corresponding brain region and sex. Multiple filled black dots connected by vertical lines represent common sets of DEGs across brain region and sex. Bar chart colors correspond to whether DEGs are shared by brain regions or sex using the bottom right key. **b**. LFC scores of all genes in the DE analysis of both brain regions clustered by cell type, brain region, and sex

We compared DEG results from the prefrontal and entorhinal cortices to determine whether changes in each sex were consistent across brain regions. Overall, we observed more overlaps across sex DEGs to be in the entorhinal cortex (Fig. 4a). Additionally, clustering samples by AD compared to control pseudobulk cell type gene expression (Fig. 4b) showed some clustering by brain region and sex.

*Pathway and Network Analysis Reveals Sex-Specific Transcriptomic Perturbations in Glial Cells in the Prefrontal Cortex and Sex-Shared, but Flipped AD-Enriched Pathways in the Entorhinal Cortex.*

**Fig. 5:**
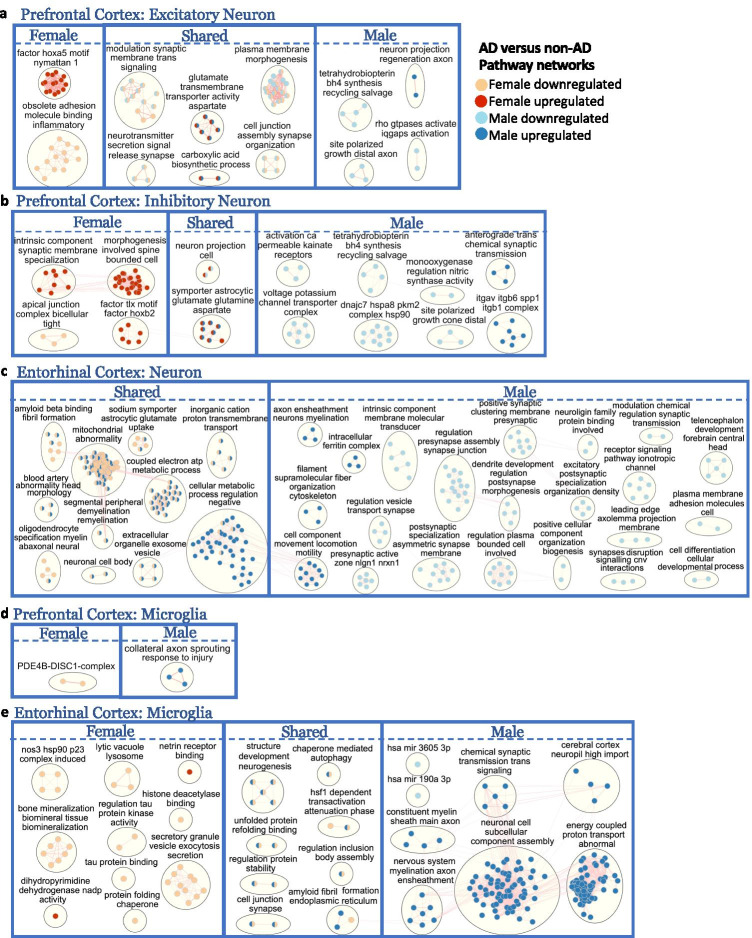
Enriched disease pathway networks in female and male neurons and microglia. AD compared to non-AD functionally enriched pathways with a BH-adjusted *p* value < 0.05 clustered into biological themes for **a**. excitatory and **b**. inhibitory neurons from the prefrontal cortex; **c**. neurons from the entorhinal cortex; and microglia from the **d**. prefrontal and **e**. entorhinal cortices. Lines represent gene set overlaps with magnitude showed by thickness

Beyond identifying sex-dimorphic disease-associated genes, we performed a gene set enrichment analysis to elucidate potential biological mechanisms implicated in disease progression that are either shared or unique to each sex and to reveal the interconnections between disease-linked pathways within AD. The pathway enrichment was performed in g:Profiler [48], a web tool that performs functional enrichment analysis from a given gene list, using separate lists of upregulated and downregulated DEGs with an adjusted *p* value < 0.05 and relaxed absolute LFC above 0.1 in cell types of each sex as inputs. Significantly enriched biological pathways with an adjusted *p* value < 0.05 were applied to EnrichmentMap [49], a functional category grouping method from the Cytoscape software, to identify pathway network clusters annotated by associated biological processes (Fig. 5, Supplementary Figs. 3 and 4).

Female and male AD compared to control excitatory neurons of the prefrontal cortex shared six common enriched clusters of pathways (Fig. 5a), which were all perturbed in the same direction for both sexes. Two of these clusters (neurotransmitter glutamate/aspartate transmembrane activity and carboxylic acid biosynthetic process) were upregulated in disease in both sexes. Of the four downregulated pathway clusters, three were related to synaptic activity (modulation of the synaptic membrane, neurotransmitter release, and synapse assembly/cell junction organization), indicating a dysregulation of synaptic plasticity in AD excitatory neurons. The other downregulated pathway cluster was plasma membrane morphogenesis, which consisted of pathways including axonogenesis, cellular projection, and plasma membrane organization (Supplementary Tables 7 and 8).

In prefrontal cortex excitatory neurons, we also identified uniquely enriched disease pathway clusters for each sex (Fig. 5a). Female excitatory neurons showed upregulation of the HOXA5 factor, a DNA-binding transcription factor that regulates cell morphogenesis and tumor suppressor that inhibits proliferation and induces apoptosis [62], and downregulation of inflammatory-mediated cell to cell interaction through adhesion and molecule binding. Interestingly, a recent epigenome-wide association study examining samples in the prefrontal cortex and superior temporal gyrus observed elevated DNA methylation of the *HOXA* gene cluster to be associated with neuropathology in AD [63]. In male excitatory neurons, we observed upregulation of axon regeneration and downregulation of distal axonal growth cone polarization. Interestingly, we also observed downregulation of tetrahydrobiopterin (BH4) synthesis, which is important for the production of essential neurotransmitters [64], and Rho GTPase activities in male AD compared to control excitatory neurons. Overall, excitatory neurons of the prefrontal cortex shared most case versus control differentially enriched pathways between male and females, the majority of which were downregulated in AD.

Like the enriched pathways in disease observed in excitatory neurons, the inhibitory neurons of the prefrontal cortex showed upregulation for glutamate/aspartate activities in both female and male AD inhibitory neurons compared to controls (Fig. 5b). Like male AD excitatory neurons, male AD inhibitory neurons also showed downregulation of axonal growth cone polarization and BH4 activities compared to controls. In addition, males specifically demonstrated upregulation in anterograde synaptic transmission and downregulation of nitric synthase, heat shock protein 90 (HSP90) complex, voltage potassium transporter, and kainite calcium-permeable receptor activities in AD. The *ITGAV-ITGB-SPP1* complex, with known function in cell adhesion [65] and without previous links to AD, was uniquely upregulated in male inhibitory neurons. Of note, the pathway cluster neuronal projection was upregulated in females and downregulated in males, consistent with the enriched upregulated pathway clusters uniquely observed in females, which were modulation of spine morphogenesis and synaptic membranes. Lastly, the transcription factors, nuclear receptor TLX (essential for the regulation of self-renewal, neurogenesis, and maintenance in neuron stem cell) [66] and nuclear protein HOXB2 (involved in cellular development) [67], were upregulated only in AD female inhibitory neurons.

Unlike in neurons in the prefrontal cortex, we identified a variety of commonly enriched disease pathway networks in entorhinal cortex neurons that were regulated in opposite directions for the sexes (Fig. 5c). For instance, amyloid-beta binding/fibril formation, mitochondrial abnormality, coupled electron ATP metabolic process, demyelination/remyelination, cellular metabolism, extracellular organelle exosome vesicle, and cation transmembrane transport were among the clusters downregulated in females and upregulated in males. We did not observe any pathway networks unique to female neurons; however, for the AD male neurons in the entorhinal cortex, we identified pathways in maintaining cellular metabolism and homeostasis, through the upregulation of genes involved in axon myelination, regulation of the metabolic process, cell component locomotion, cytoskeleton organization, and intracellular ferritin complex (iron storage). In male neurons, we also observed synaptic activity deficiency, indicated by the downregulation of pathways in synaptic vesicle transport, presynaptic assembly at cell junction, synaptic membrane clustering, postsynaptic membrane morphogenesis, chemical regulation at the synapse, neuroligin family protein binding, and ionotropic receptor signaling. Additionally, male AD neurons compared to controls also showed downregulation in plasma membrane regulation, cell projection, and developmental process in differentiation. While sex differences are minimal in the neurons of the prefrontal cortex, we observed overwhelmingly shared but inversely regulated enrichment pathways in the neurons of the entorhinal cortex.

Microglia, the resident immune cells of the brain, have gained growing recognition as being critically involved in AD pathogenesis due to their key role contributing to neuroinflammation, a prominent feature of AD [68]. Only a few significantly enriched disease pathways were observed in microglial cells of the prefrontal cortex, and none was shared across sexes (Fig. 5d). We observed upregulation of axon sprouting in response to injury in males, as well as an enriched upregulated pathway in axonogenesis regulation in females (Supplementary Table 8). Interestingly, a cluster of the *PDE4B-DISC1* complex, with important functions in cAMP-regulated signal transduction and synaptic plasticity [69], was downregulated in females. The phosphodiesterase 4B (PDE4B) enzyme was previously shown to be pro-inflammatory in microglia and is currently under study as a therapeutic target for neuroinflammation and cognitive function impairment [69].

Microglia in the entorhinal cortex had mostly downregulated pathway clusters in females and upregulated pathway clusters in males (Fig. 5e). Amyloid fibril formation, chaperone-mediated autophagy, protein folding, protein stability regulation, cell junction synapse, neurogenesis structure development, and cell body assembly were among the clusters shared by both sexes but downregulated in females and upregulated in males. Protein homeostasis was altered in disease for females, as shown by downregulation of tau protein kinase activity, tau protein binding, protein folding chaperone, and histone deacetylase binding. Protein degradation and secretion were also downregulated in females with AD compared to controls, as indicated through downregulation of lytic vacuole lysosome and secretory granule vesicle exocytosis respectively. Interestingly, nitric oxide synthase 3 (NOS3), which is involved in a complex cascade of events in oxidative stress that may induce cellular injury and accelerate neurodegenerative changes [70], and its chaperone, HSP90 [71], were downregulated in AD females compared to controls. In males, myelination in axon ensheathment, synaptic signaling transmission, and energy-coupled proton transport were upregulated. We also identified downregulation of two microRNA clusters, hsa-miR-190a and hsa-miR-3605, in AD males compared to healthy controls. These are potentially important findings because epigenetic modulation by microRNAs has the capacity to modify microglial behavior in physiological conditions, and dysregulation of microRNAs could mediate microglial hyper-activation and persistent neuroinflammation in neurological diseases [72]. Overall, we observed extensive sex-specific pathway enrichments in microglial populations of AD compared to controls for both brain regions, but especially pronounced in entorhinal cortex.

Furthermore, astrocytes, oligodendrocytes, and OPCs also demonstrated sex-specific pathway perturbations in both prefrontal and entorhinal cortices (Supplementary Figs. 3 and 4). In astrocytes, which normally function to maintain overall brain homeostasis, we observed downregulated plasma and presynaptic membrane components and upregulated postsynaptic asymmetric synapse density in the prefrontal cortex of AD compared to controls in both sexes. In female AD astrocytes, we observed downregulation in pathways related to amino acid transport and vascular transport across the blood–brain barrier. Although the downregulation of these pathways was not observed in males, a related pathway cluster, presynaptic filopodia activities, was downregulated. These observed pathway networks suggest that the same biological process, regulation of synaptic activities, was disrupted in both sexes but via different mechanisms.

In oligodendrocytes, which provide support and insulation to axons in the brain, we observed downregulation in pathways related to regulation of synaptic activity in both female and male AD compared to controls, indicated by the downregulated clusters of cleft regulation, presynaptic assembly, and transmembrane transport channel in females, and neurotransmitter secretion, transmembrane ion transporter, and postsynaptic membrane potential regulation in males. Interestingly, pathways related to cell morphological changes and energy production were upregulated in males and downregulated in females, such as pathway clusters of neuron projection organization, cell migration/locomotion, cellular component organization, ATP coupled electron transport, and mitochondrial NADH dehydrogenase, suggesting oligodendrocyte responses were sex-specific when challenged by disease.

Lastly, we observed upregulation of membrane morphogenesis in female OPCs in the prefrontal cortex, as well as related pathway cluster, *TROY-NGR-LINGO1-NGFR* complex, which plays essential roles in the inhibition of axonal regeneration [73]. In the entorhinal cortex, a few pathways were downregulated in female and male OPCs, including cell junction synapse assembly, glutamatergic synapse, and plasma membrane intrinsic component. The male OPCs of the entorhinal cortex were overwhelmingly enriched with upregulation in neuronal development, axon ensheathment, neuron myelination, and metabolic protein regulation, as well as ion and vesicle transport, with the exception that synaptic membrane adhesion molecules were downregulated. Although inconclusive due to the unbalanced numbers of significantly enriched pathways obtained in OPCs from both sexes, our observations suggest that AD female OPCs in the prefrontal cortex diverge more from controls compared to male OPCs, whereas in the entorhinal cortex, AD male OPCs were more perturbed by disease status compared to females.

## Discussion

Men and women show differing vulnerabilities to AD, with increased longevity and prevalence in women, and decreased tau and possibly cognitive decline in men [17–19, 21, 22]. To understand how AD presents in each sex on a cell type-specific level, we performed a sex-stratified differential gene expression (DGE) and pathway network analysis on the five main brain cell types using the first two publicly available human single-nucleus RNA-Seq datasets. The two datasets target two separate brain regions, the entorhinal and prefrontal cortices, and we analyzed each in a sex-stratified manner, then compared findings across sexes and brain regions to highlight both general and cell type-, region-, and sex-specific transcriptional phenotypes of AD (Fig. 1).

Our gene-level analysis in the prefrontal cortex demonstrated sex-distinct disease-related transcriptomic changes when comparing their respective cases to controls (Fig. 2). We observed more DEGs shared among cell types in females versus males (e.g., *LINGO1* [50, 51], *SLC1A3* [52], *SPP1* [53]) and a larger range of fold change in our female DGE analysis. Additionally, through clustering prefrontal cortex samples based on AD compared to control pseudobulk gene expression, we observed samples to cluster first by sex in all cell types except excitatory neurons. We observed *LINGO1*, a negative modulator of neuronal survival and axonal integrity [50], to be upregulated in AD females but not in AD males. Besides being part of the *TROY-NGR-LINGO1-NGFR* complex, which inhibits neuronal growth cones and myelination, Lingo1 was also found to bind directly to APP and facilitate access to β-secretase and inhibit α-secretase cleavage which leads to increased production of Aβ fragments [51]. Higher levels of *LINGO1* in females might contribute to neuronal death and Aβ accumulation. We also observed upregulation of *SPP1*, which encodes the protein osteopontin [53], in the excitatory neurons and microglia in both females and males, as well as inhibitory neurons and astrocytes in females. *SPP1* was previously shown to play a pro-inflammatory role in the presence of aggregated tau and *SPP1* knockout mice resulted in reduced neuroinflammation following an inflammatory challenge with lipopolysaccharide (LPS) [74]. This suggests that AD females might experience slightly elevated neuroinflammation and increased vulnerability to tau toxicity.

In the entorhinal cortex, compared to the prefrontal cortex, we observed more DEGs and many global changes across cell types of both sexes (Fig. 3a). Through clustering entorhinal cortex samples by AD compared to control pseudobulk gene expression, we observed samples to cluster by sex for all cell types and observed opposing expression patterns across sex (Fig. 3b–d), implying sex-distinct mechanisms of neurodegeneration in the entorhinal cortex. Surprisingly, entorhinal cortex samples reveal upregulation of *LINGO1* in all cell types of AD males but only in astrocytes of AD females. We hypothesize that the variable enrichment findings in the prefrontal versus entorhinal cortices might have resulted from sex dimorphic temporal progression of the disease pathology across brain regions. Further work is required to understand the role of *LINGO1* in AD. We also observed upregulation of *CST3* in neurons and oligodendrocytes of AD males but downregulation in AD females. Cystatin C (CysC), encoded by *CST3*, was previously linked to AD and shown to bind Aβ and inhibit Aβ oligomerization and fibril formation [75]. Studies suggest that CysC is neuroprotective and reduced levels of CysC increase neuronal vulnerability to insults and neurodegeneration. Our observation of the sex dimorphic expressions of *CST3* suggests that AD females are more vulnerable to AD pathology. Moreover, our comparative analysis across brain regions showed more DEG overlaps across sex in the entorhinal cortex and disease-related changes of gene expression to be influenced by brain region and sex (Figs. 3 and 4).

From the gene-set enrichment and pathway clustering network analysis, we identified sex-specific pathway network changes, which are potentially involved in AD pathogenesis through mechanisms unique to each sex (Fig. 5, Supplementary Fig. 3 and Supplementary Fig. 4). Our results demonstrated that diseased neurons in the prefrontal cortex shared more enriched pathways compared to glial cells in both sexes, indicated by the proportion and directionality of the shared pathways. This may suggest that neuronal pathophysiology is similar in female versus male, and glial pathophysiological changes are more distinctive in contributing to sex-specific disease progression in AD. Despite neurons being more similar than glial cells, interesting sex-specific biological perturbations were revealed in neurons of females and males separately. Diseased female neurons showed increased activation in cell membrane morphogenesis but reduction in the production of tight junction complexes. A few transcriptional factors were uniquely upregulated in females, such as HOXA5, HOXB2, and TLX. Future studies investigating the role of overactivation of these genes in AD, especially in females, could lead to better mechanistic understanding of AD pathogenesis and potential therapies targeting these transcriptional factors in females. In diseased male neurons, nitric oxide synthase (NOS) activity was downregulated, as well as its regulating factors, the HSP90 complex and cofactor BH4. BH4 has been extensively studied in its role of regulating nitric oxide production from nitric oxide synthases and superoxide anion radical (O_2_^•−^) release in the endothelium [76]. Our pathway enrichment analysis suggests that perhaps excessive O_2_^•−^ in diseased male neurons due to dysregulated NOS activities and BH4 levels could lead to neuronal stress and death. Therefore, resolving the chronic BH4 deficiency and change in redox state of neurons pharmacologically could be a beneficial therapy for AD male patients.

The glial cells in the prefrontal cortex shared just a few enriched pathways, out of hundreds detected collectively, between AD males and females: nine in astrocytes (Supplementary Fig. 3a), two in oligodendrocytes (Supplementary Fig. 4a), and none in microglia and OPCs (Fig. 5d, Supplementary Fig. 4c) (these numbers do not include shared pathways regulated in opposite directions in female versus male). Besides downregulation of membrane morphogenesis, both female and male diseased astrocytes demonstrated decreased synaptic regulation, but different pathways for different components were involved. In females, we observed a decrease in glutamate transmembrane transport, vascular transport, and organic acid symporter activities. In males, we observed a decrease in presynaptic intrinsic component filopodia activities. These pathways are interconnected, indicating that they belong to related biological processes, which suggests that similar resulting synaptic deficiencies were observed in both sexes but resulted from different pathway mechanisms. These present compelling evidence for focusing on glial cell pathophysiological changes in studying sex differences in AD pathogenesis.

In the entorhinal cortex, while like in the prefrontal cortex, we identified sex-specific perturbed pathway networks in all cell types, where the pathways shared across sexes were overwhelmingly of opposite direction, with most pathways downregulated in female and upregulated in males (Fig. 5, Supplementary Fig. 3 and Supplementary Fig. 4). Out of the five cell types investigated, two were dominated by enriched pathways detected in males (neurons and OPCs), one was dominated by enriched pathways detected in females (oligodendrocytes), and two were more evenly distributed (microglia and astrocytes). The diseased female microglia demonstrated deficiency in tau protein processing uniquely, by downregulation of tau kinase activity and tau protein binding. Additionally, disruption of cellular protein homeostasis was also observed in female microglia, indicated by downregulation of protein folding chaperone, histone deacetylase binding, lysosomal activity, and exocytosis vesicle secretion. The female microglia were perceived as deficient in dealing with the degradation of the debris and cellular waste that they phagocytosed while the male microglia were active at combating the disease environment by upregulating axonal myelination, synaptic transmission signaling, cellular component assembly, and energy production through energy-coupled proton transport. As immune cells are critical for repair after injury, this may indicate that female AD risk relates to decreased ability to properly recover after deleterious events over time.

While we observed evidence of sex-dimorphic disease changes in glial cells in AD, it is important to note some limitations in the study. First, the datasets were limited in sample size. The entorhinal cohort consisted of six cases (two females, four males) and five controls (two females, three males) (Fig. 1, Table 2, Supplementary Table 2), while the prefrontal cohort consisted of 20 cases (10 females, 10 males) and 22 controls (10 females, 12 males) (Fig. 1, Table 1, Supplementary Table 1). While the number of samples is low as mentioned above, the analysis is carried out on the level of cells and there are still a significant number of cells available for each comparison which gives us the power to detect robust differential expression signals, but future studies characterizing expression across more samples on a single-cell level will allow for more robust analysis. Second, there were batch effects in the entorhinal cortex data introduced by the study design. This was overcome by performing data integration and including *APOE* genotype as a covariate in our DGE analysis to account for batch and avoid collinearity in our model. Next, although both datasets were age-matched, they were not *APOE* genotype matched. APOE4 is the largest risk factor in AD, and as a result, we would expect some transcriptional differences based on the *APOE* genotype of a sample [77]. In the prefrontal cortex cohort, female samples had cases but not controls with the ε4 allele of *APOE*, and male samples had cases and only one control sample with ε4 allele of *APOE* (Table 1)*.* In the entorhinal cortex cohort, female samples included one of two cases and no controls with an ε4 allele of *APOE*, and all male cases had at least one ε4 allele of *APOE*, and one of three control samples had an ε4 allele of *APOE* (Table 2, Supplementary Table 2). While we accounted for *APOE* genotype as a covariate in the DGE analysis, the interactions of sex and *APOE* genotype may still explain trends that we observe.

Additionally, interpretation at the DEG level (Figs. 2a, 3a, and 4a) was limiting without disease-relevant mechanistic insights. We observed genes involved in tau protein binding and regulation of tau-protein kinase activity, such as *HSP90AA1* [78] and *CLU* [79], to be downregulated in AD female microglia, suggesting higher vulnerability to tau toxicity in AD females. The downregulated genes in the entorhinal cortex in females are potentially related to tau deposition in this area, but inconclusive to suggest any causal association since AD cases were grouped based on Braak staging, which measures the severity of tau deposition. AD females and males have similar tau pathology in this analysis, and stratification by tau levels was considered but not feasible due to the small sample size (number of patients).

The accumulation of sex dimorphic pathophysiological depositions in AD brains during aging may be influenced by sex-specific hormones. Although the exact mechanisms remain elusive, neuroactive steroids have been shown to be anti-inflammatory and neuroprotective by alleviating mitochondrial dysfunction (26). Evidence indicates that many neurosteroids function to increase ATP production and restore mitochondrial membrane potential, with estrogen and progesterone being more effective in AD-related tauopathy models and testosterone being more robust in a model of Aβ-induced mitochondrial deficits [76]. Furthermore, estrogen and progesterone have beneficial effects of reducing tau phosphorylation through specific receptor binding in neurons to activate signal transduction and inhibit tau kinase activities. These hormones play a critical role in sex dimorphic tau content in the brain. A study showed that total tau level decreases and the ratio of phosphorylated tau increases in the hippocampus of rats during pregnancy [80], while hormone levels fluctuate dramatically. The interplay between estrogen and progesterone and their interaction with Aβ and tau might be involved in the sex dimorphic vulnerability to Aβ and tau toxicity.

To summarize novel and previously studied DEGs in AD, we extended our analysis to include pathway and network enrichment. When comparing our results across brain regions, noting the limitations of each dataset, we were cautious to not further explore unique molecular profiles in each region, which could give insight into the spread of AD pathology as it relates to sex. Moreover, literal biological sex could be a misleading classifier for trans* individuals. A properly powered study of differences between male versus female versus recipients of testosterone- versus estrogen-focused hormone replacement therapy might help narrow down a genetic versus hormonal basis of DEGs deemed sexually dimorphic. Overall, we hope that larger and different types of omics datasets from more brain regions of individuals with diverse age groups, racial and ethnic backgrounds, and *APOE* genotypes become available to allow for future explorations of sex-specific multiomic changes in AD that will address these points.

## Conclusions

In general, our findings suggest that AD signatures in neurons in the prefrontal cortex were more similar in females and males compared to glial cells, as indicated by the proportions of sex-shared genes and pathways with directionally similar regulation in each cell type (Fig. 5, Supplementary Fig. 3 and Supplementary Fig. 4). In the entorhinal cortex, while we identified sex-specific perturbed pathways in each cell type, the sex-shared pathways were overwhelmingly opposite in the direction of regulation, with most pathways downregulated in female and upregulated in males or conversely regulated for a few other pathways, suggesting differential mechanisms of neurodegeneration between sexes. Sex-stratified findings in the entorhinal cortex could relate to recent observations that women show more tau deposition early on in the AD trajectory, specifically in this area [81]. Perhaps future studies could also explore the specific association between the gene changes in the entorhinal region with tau burden. Collectively, these observed sex-specific transcriptomic changes provide a valuable resource to study sex-specific cell type-specific pathophysiology of AD. Although expression differences in all cell types may be relevant to disease mechanisms in AD, we focused on discussing the cell types with the most compelling findings in our study: neurons, astrocytes, and microglia. We hope this work serves as a resource for follow-up studies that will examine more deeply all the cell types and their specific roles leading to sex-specific AD pathophysiology.

## Supplementary Information

Below is the link to the electronic supplementary material.

Supplementary file1
Additional file 1 (.xls)
Supplementary Table 3: Prefrontal cortex cell type composition
Supplementary Table 4: Entorhinal cortex cell type composition
Supplementary Table 5: Prefrontal cortex differentially expressed genes
Supplementary Table 6: Entorhinal cortex differentially expressed genes
Supplementary Table 7: Enriched disease pathways in female cells in the Prefrontal cortex
Supplementary Table 8: Enriched disease pathways in male cells in the Prefrontal cortex
Supplementary Table 9: Enriched disease pathways in female cells in the Entorhinal cortex
Supplementary Table 10: Enriched disease pathways in male cells in the Entorhinal cortex (XLS 1512 KB)

Supplementary file2 Additional file 2 (.pdf): Supplementary Figure 1: Dimensionality reduction of prefrontal and entorhinal cortices cohort cells by covariates. APOE genotype, batch, cell type, diagnosis, and sex represented in the a. prefrontal cortex, b. entorhinal cortex before batch correction, and c. entorhinal cortex after batch correction. (PDF 5805 KB)

Supplementary file3 Additional file 3 (.pdf): Supplementary Figure 2: Shared and unique disease signatures across and within sex, cell types, and brain regions. Upset plots indicating intersections of AD versus non-AD DEGs (BH adjusted p-value < 0.05 and absolute LFC > 0.25). Rows correspond to cell type, direction of gene expression change, and sex in respective plots. The bar chart shows the number of single and common DEGs. Single filled dots represent a unique set of DEGs, and multiple filled black dots connected by vertical lines represent common DEGs. (PDF 300 KB)

Supplementary file4 Additional file 4 (.pdf): Supplementary Figure 3: Enriched disease pathway networks in female and male astrocytes. AD compared to non-AD functionally enriched pathways with a BH adjusted p-value < 0.05 clustered into biological themes for astrocytes in a. prefrontal, and b. entorhinal cortices. Lines represent gene set overlaps with magnitude showed by thickness.(PDF 9575 KB)

Supplementary file5 Additional file 5 (.jpeg): Supplementary Figure 4: Enriched disease pathway networks in female and male oligodendrocytes and OPCs. AD compared to non-AD functionally enriched pathways with a BH adjusted p-value < 0.05 clustered into biological themes for a, b. oligodendrocytes and c, d. OPCs in prefrontal and entorhinal cortices. Lines represent gene set overlaps with magnitude showed by thickness. (JPG 373 KB)

## Data Availability

We accessed single-nuclei RNA-Seq count data from the prefrontal cortex via the Accelerating Medicines Partnership Alzheimer’s Disease Project (AMP-AD) Knowledge Portal under the Religious Orders Study and Memory and Aging Project (ROSMAP) (https://www.synapse.org/#!Synapse:syn18485175; https://www.synapse.org/#!Synapse:syn3157322) and from the entorhinal cortex via a data repository provided by Grubman et al. (http://adsn.ddnetbio.com/). The entorhinal cortex dataset and supporting materials may also be accessed via the Gene Expression Omnibus (GEO) under the accession number GSE138852. Access to the prefrontal cortex dataset requires a formal request to ROSMAP. All codes necessary for recreating the reported analyses and figures within R are available at https://github.com/stebel5/AD_SexDiff_snRNAseq.

## Abbreviations

AD: Alzheimer’s disease
Aβ: Amyloid-β
*APOE*: Apolipoprotein E
RNA-Seq: RNA sequencing
DGE: Differential gene expression
CERAD: Consortium to Establish a Registry for Alzheimer’s Disease
PCA: Principal component analysis
UMAP: Uniform Manifold Approximation and Projection
BH: Benjamini-Hochberg
DEG: Differentially expressed gene
OPC: Oligodendrocyte progenitor cells
