# Supplementary figures and images for: Sex-Stratified Single-Cell RNA-Seq Analysis Identifies Sex-Specific and Cell Type-Specific Transcriptional Responses in Alzheimer’s Disease Across Two Brain Regions
Source: Mol Neurobiol. 2021 Oct 20;59(1):276–93. doi: 10.1007/s12035-021-02591-8 (PMC8786804; doi:10.1007/s12035-021-02591-8)

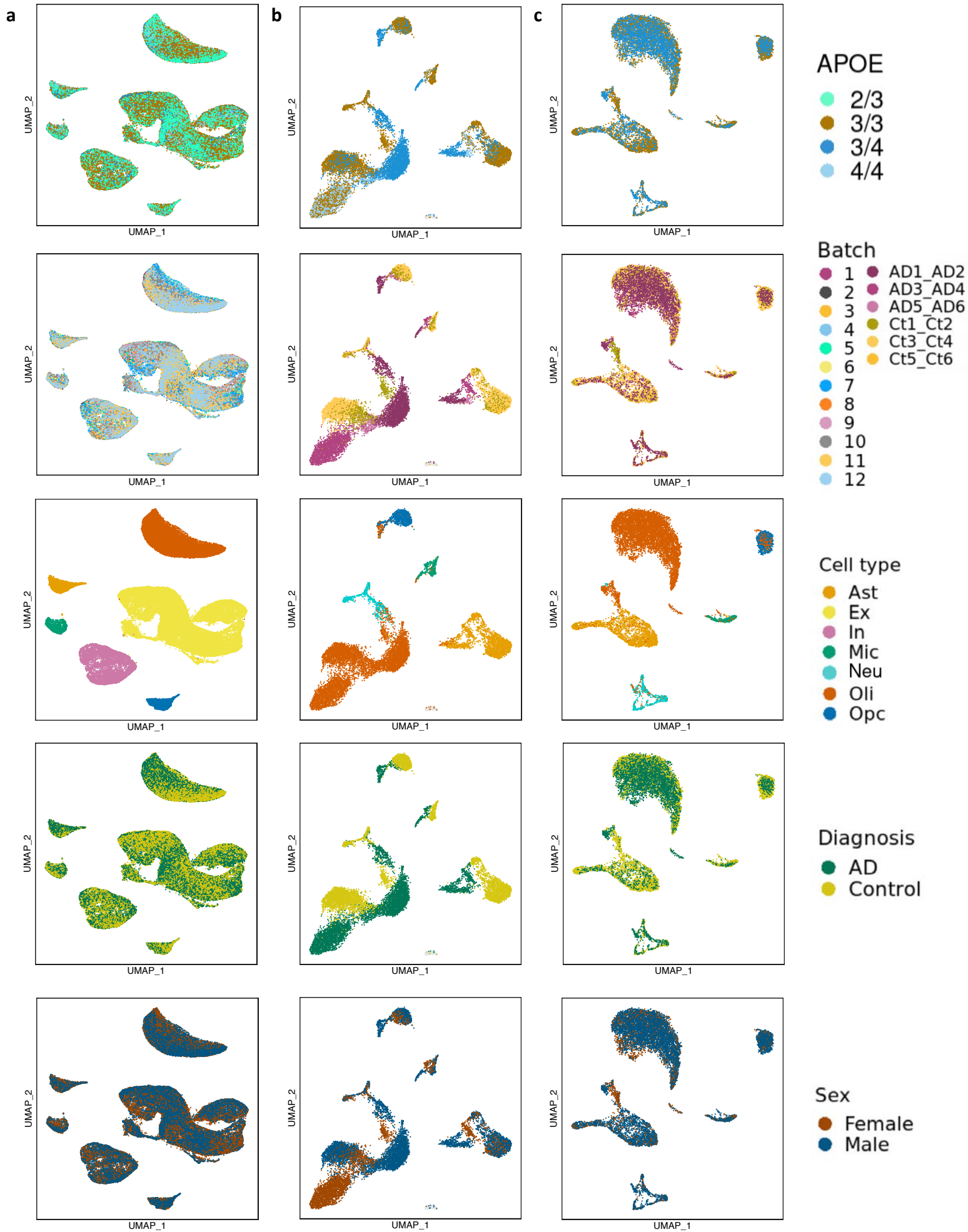

Supplement: Supplementary file 2 — Supplementary file2 Additional file 2 (.pdf): Supplementary Figure 1: Dimensionality reduction of prefrontal and entorhinal cortices cohort cells by covariates. APOE genotype, batch, cell type, diagnosis, and sex represented in the a. prefrontal cortex, b. entorhinal cortex before batch correction, and c. entorhinal cortex after batch correction. (PDF 5805 KB) [file 12035_2021_2591_MOESM2_ESM.pdf]

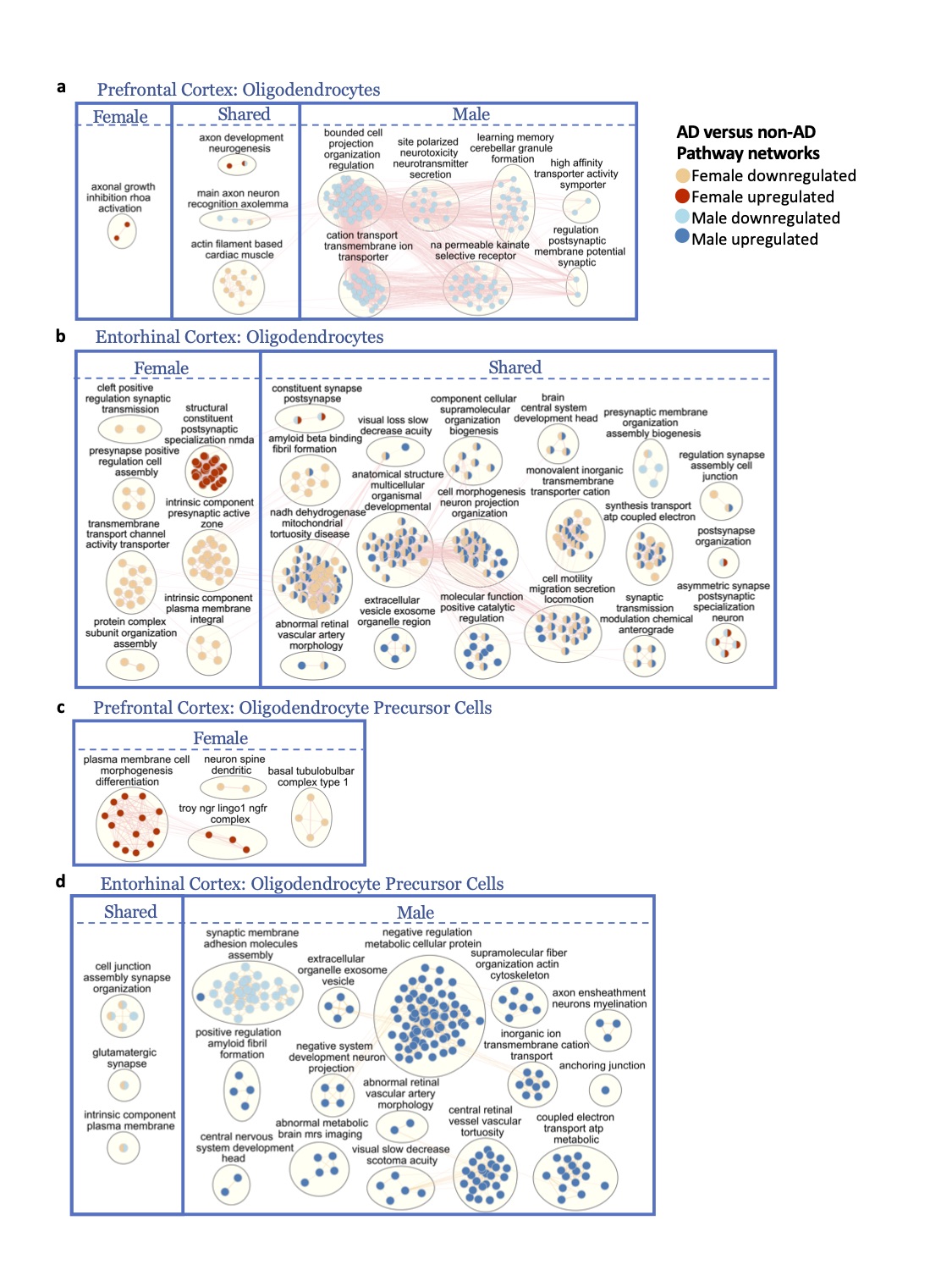

Supplement: Supplementary file 5 — Supplementary file5 Additional file 5 (.jpeg): Supplementary Figure 4: Enriched disease pathway networks in female and male oligodendrocytes and OPCs. AD compared to non-AD functionally enriched pathways with a BH adjusted p-value < 0.05 clustered into biological themes for a, b. oligodendrocytes and c, d. OPCs in prefrontal and entorhinal cortices. Lines represent gene set overlaps with magnitude showed by thickness. (JPG 373 KB) [file 12035_2021_2591_MOESM5_ESM.jpg]
