# Supplementary material for: Sex-Stratified Single-Cell RNA-Seq Analysis Identifies Sex-Specific and Cell Type-Specific Transcriptional Responses in Alzheimer’s Disease Across Two Brain Regions
Source: Mol Neurobiol. 2021 Oct 20;59(1):276–93. doi: 10.1007/s12035-021-02591-8 (PMC8786804; doi:10.1007/s12035-021-02591-8)

Shared and unique male and female disease signatures across cell types in the prefrontal cortex

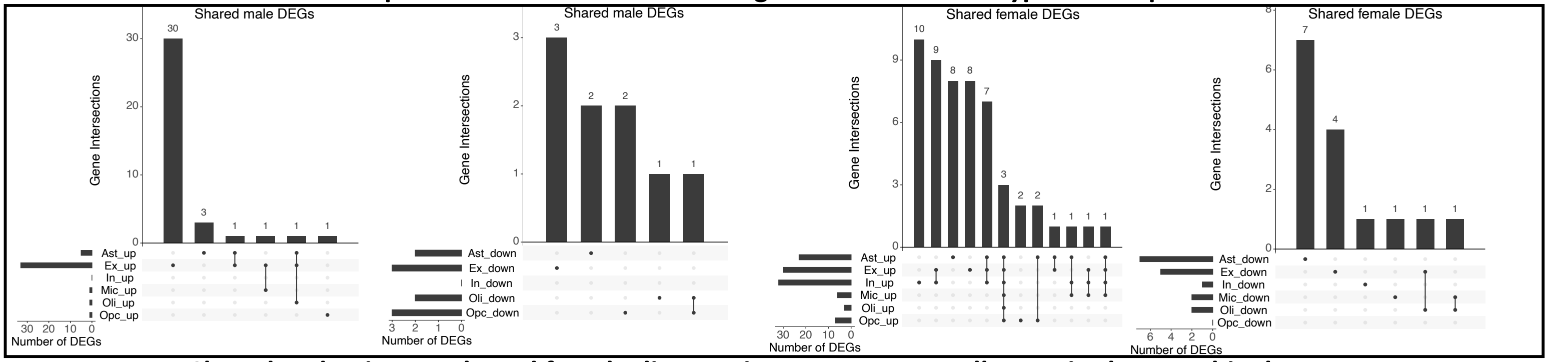

Shared and unique male and female disease signatures across cell types in the entorhinal cortex

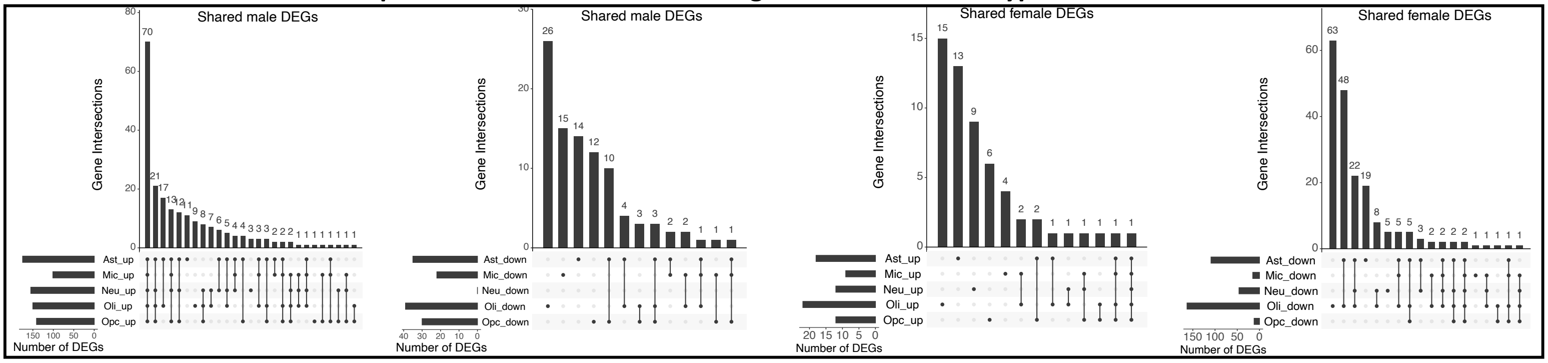

Shared and unique male and female disease signatures within cell types in the prefrontal cortex

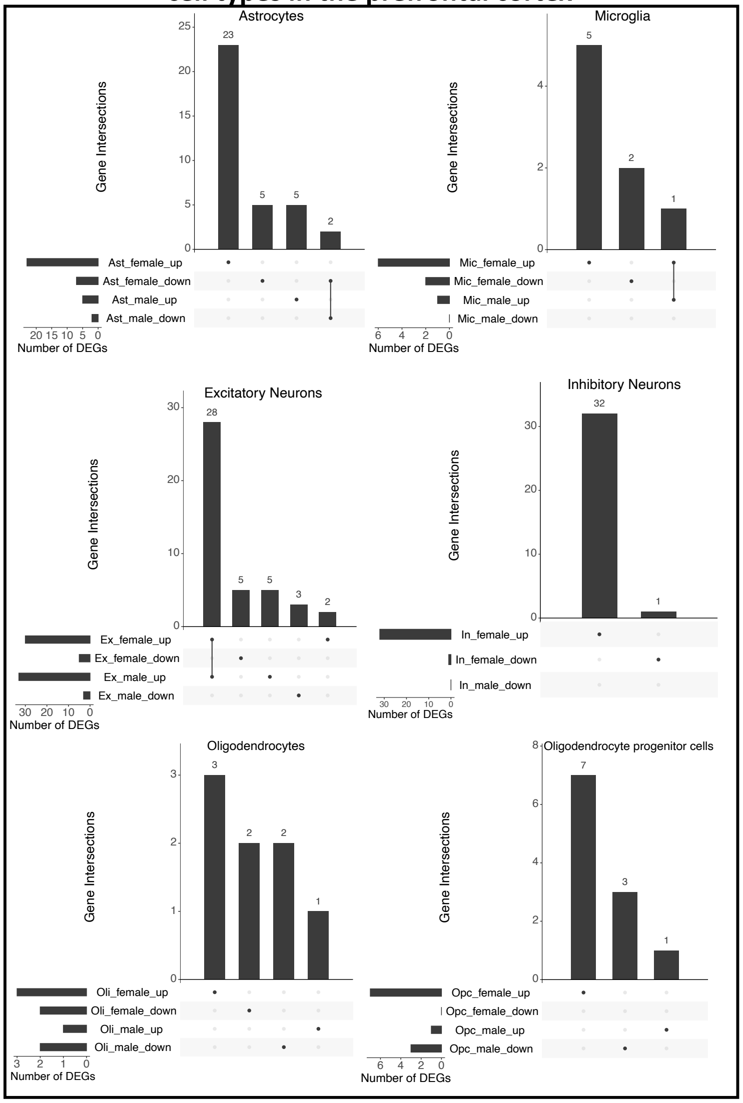

Shared and unique male and female disease signatures within cell types in the entorhinal cortex

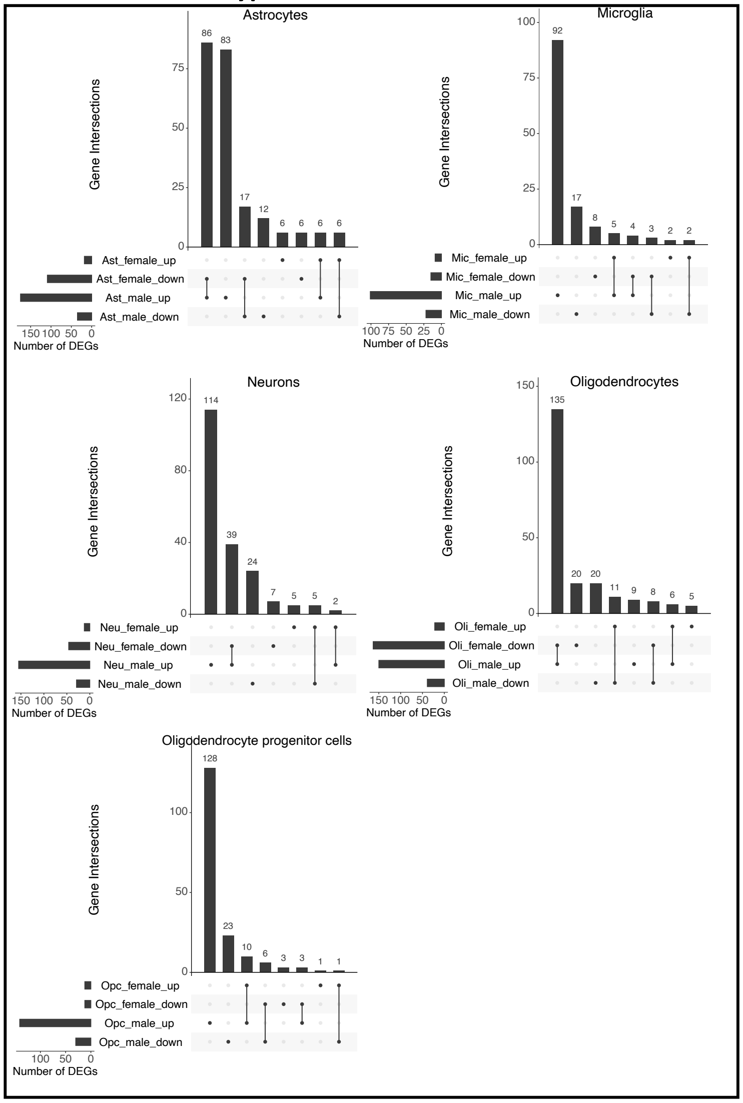

Supplement: Supplementary file 3 — Supplementary file3 Additional file 3 (.pdf): Supplementary Figure 2: Shared and unique disease signatures across and within sex, cell types, and brain regions. Upset plots indicating intersections of AD versus non-AD DEGs (BH adjusted p-value < 0.05 and absolute LFC > 0.25). Rows correspond to cell type, direction of gene expression change, and sex in respective plots. The bar chart shows the number of single and common DEGs. Single filled dots represent a unique set of DEGs, and multiple filled black dots connected by vertical lines represent common DEGs. (PDF 300 KB) [file 12035_2021_2591_MOESM3_ESM.pdf]
