# Supplementary material for: Sex-Stratified Single-Cell RNA-Seq Analysis Identifies Sex-Specific and Cell Type-Specific Transcriptional Responses in Alzheimer’s Disease Across Two Brain Regions
Source: Mol Neurobiol. 2021 Oct 20;59(1):276–93. doi: 10.1007/s12035-021-02591-8 (PMC8786804; doi:10.1007/s12035-021-02591-8)
Supplement: Supplementary file 4 — Supplementary file4 Additional file 4 (.pdf): Supplementary Figure 3: Enriched disease pathway networks in female and male astrocytes. AD compared to non-AD functionally enriched pathways with a BH adjusted p-value < 0.05 clustered into biological themes for astrocytes in a. prefrontal, and b. entorhinal cortices. Lines represent gene set overlaps with magnitude showed by thickness.(PDF 9575 KB) [file 12035_2021_2591_MOESM4_ESM.pdf]

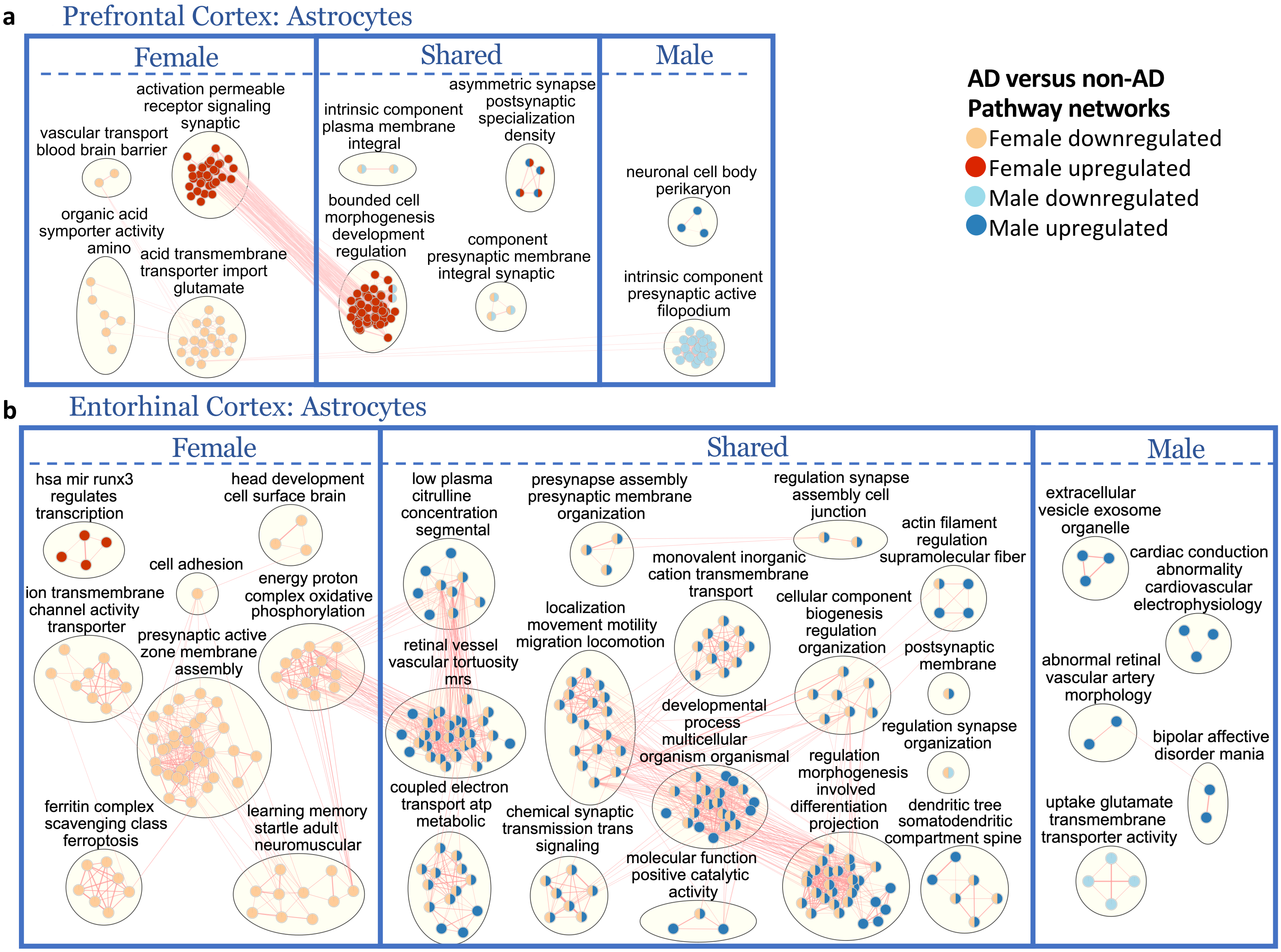
a
Prefrontal Cortex: Astrocytes
Female
Shared
Male
activation permeable receptor signaling synaptic
vascular transport blood brain barrier
organic acid symporter activity amino
acid transmembrane transporter import glutamate
intrinsic component plasma membrane integral
asymmetric synapse postsynaptic specialization density
bounded cell morphogenesis development regulation
component presynaptic membrane integral synaptic
neuronal cell body perikaryon
intrinsic component presynaptic active filopodium
AD versus non-AD Pathway networks
Female downregulated
Female upregulated
Male downregulated
Male upregulated
b
Entorhinal Cortex: Astrocytes
Female
Shared
Male
hsa mir runx3 regulates transcription
head development cell surface brain
cell adhesion
energy proton complex oxidative phosphorylation
ion transmembrane channel activity transporter
presynaptic active zone membrane assembly
ferritin complex scavenging class ferroptosis
learning memory startle adult neuromuscular
low plasma citrulline concentration segmental
presynapse assembly presynaptic membrane organization
regulation synapse assembly cell junction
actin filament regulation supramolecular fiber
monovalent inorganic cation transmembrane transport
cellular component biogenesis regulation organization
localization movement motility migration locomotion
retinal vessel vascular tortuosity mrs
postsynaptic membrane
developmental process multicellular organism organismal
regulation synapse organization
regulation morphogenesis involved differentiation projection
dendritic tree somatodendritic compartment spine
coupled electron transport atp metabolic
chemical synaptic transmission trans signaling
molecular function positive catalytic activity
extracellular vesicle exosome organelle
cardiac conduction abnormality cardiovascular electrophysiology
abnormal retinal vascular artery morphology
bipolar affective disorder mania
uptake glutamate transmembrane transporter activity
